# Supplementary material for: PCSK9 as an Atherothrombotic Risk Factor
Source: Int J Mol Sci. 2023 Jan 19;24(3):1966. doi: 10.3390/ijms24031966 (PMC9916735; doi:10.3390/ijms24031966)
Supplement: Supplementary file 1 [file ijms-24-01966-s001.zip › ijms-2165235-supplementary.pdf]

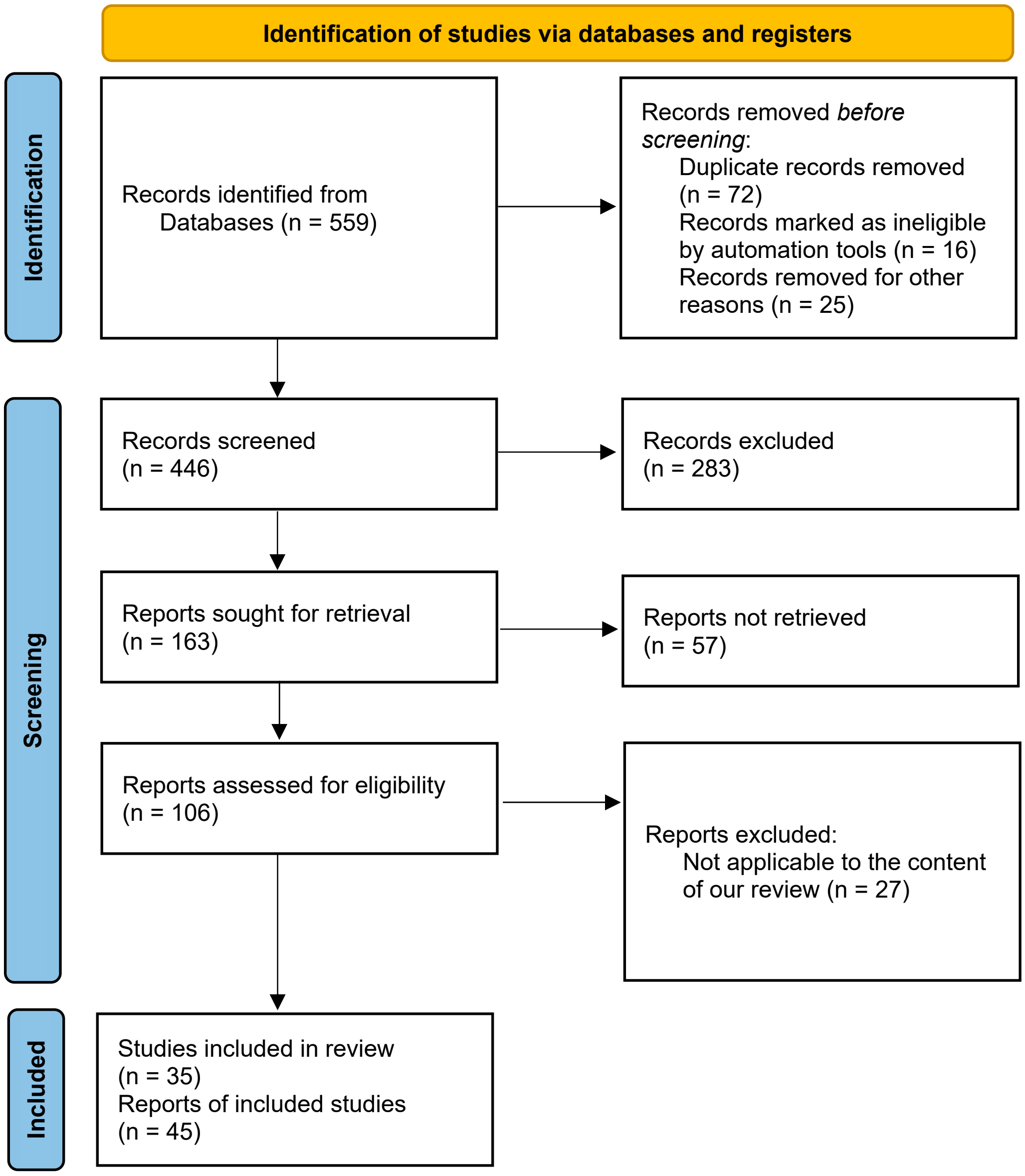

Supplementary Figure S1: PRISMA flow diagram was used to select the studies. Searches were performed in PubMed database under key words PCSK9 and coagulation, PCSK9 and fibrinolysis, PCSK9 and thrombosis, PCSK9 and inflammation, PCSK9 and platelet function.
